# Plantar Pressure-Based Gait Recognition with and Without Carried Object by Convolutional Neural Network-Autoencoder Architecture

**DOI:** 10.3390/biomimetics10020079

**Published:** 2025-01-26

**Authors:** Chin-Cheng Wu, Cheng-Wei Tsai, Fei-En Wu, Chi-Hsuan Chiang, Jin-Chern Chiou

**Affiliations:** 1 Department of Biomechatronic Engineering, National ILAN University, No. 1, Sec. 1, Shennong Rd., Yilan 26047, Taiwan; chincheng@niu.edu.tw; 2 Department of Electronics and Electrical Engineering, National Yang Ming Chiao Tung University, 1001 University Road, Hsinchu 30010, Taiwan; jwtsai.ee06@nycu.edu.tw (C.-W.T.); adidas30603.ee09@nycu.edu.tw (F.-E.W.); chiangch.ee11@nycu.edu.tw (C.-H.C.); 3 Institute of Electrical and Control Engineering, National Yang Ming Chiao Tung University, 1001 University Road, Hsinchu 30010, Taiwan

**Keywords:** plantar pressure, gait recognition, convolutional neural network-autoencoder architecture

## Abstract

Convolutional neural networks (CNNs) have been widely and successfully demonstrated for closed set recognition in gait identification, but they still lack robustness in open set recognition for unknown classes. To improve the disadvantage, we proposed a convolutional neural network autoencoder (CNN-AE) architecture for user classification based on plantar pressure gait recognition. The model extracted gait features using pressure-sensitive mats, focusing on foot pressure distribution and foot size during walking. Preprocessing techniques, including region of interest (ROI) selection, feature image extraction, and data horizontal flipping, were utilized to establish a CNN model that assessed gait recognition accuracy under two conditions: without carried items and carrying a 500 g object. To extend the application of the CNN to open set recognition for unauthorized personnel, the proposed convolutional neural network-autoencoder (CNN-AE) architecture compressed the average foot pressure map into a 64-dimensional feature vector and facilitated identity determination based on the distances between these vectors. Among 60 participants, 48 were classified as authorized individuals and 12 as unauthorized. Under the condition of not carrying an object, an accuracy of 91.218%, precision of 93.676%, recall of 90.369%, and an F1-Score of 91.993% were achieved, indicating that the model successfully identified most actual positives. However, when carrying a 500 g object, the accuracy was 85.648%, precision was 94.459%, recall was 84.423%, and the F1-Score was 89.603%.

## 1. Introduction

The gait recognition system has been extensively researched across various domains, particularly in the analysis of gait under different pathological conditions, including patients undergoing dialysis, individuals with Parkinson’s disease, and frail elderly patients in hospital environments [1,2]. By systematically collecting gait data from these populations, healthcare professionals can achieve effective classification and diagnosis, thereby facilitating the development of targeted treatment plans.

For the elderly, the risk of falls significantly increases, which may lead to prolonged bed rest and subsequent disability [3]. The implementation of gait analysis technology enables early prediction of fall risks and the initiation of preventive measures. Furthermore, gait recognition systems play a crucial role in evaluating rehabilitation outcomes for stroke patients and individuals recovering from orthopedic surgeries. By analyzing changes in patients’ gait patterns, clinicians can make more precise adjustments to rehabilitation protocols, ultimately enhancing recovery outcomes. For patients with chronic conditions such as hypertension and diabetes, gait analysis serves as a valuable health monitoring tool that aids in the early detection of declining walking abilities, thus preventing falls and other adverse events [2]. Additionally, gait recognition systems can function as auxiliary diagnostic tools, assisting clinicians in swiftly identifying and assessing movement-related disorders such as Parkinson’s disease [1]. Through automated data analysis, healthcare providers can more effectively evaluate patients’ health statuses.

The integration of gait recognition technology with smart home systems contributes to the monitoring and care of elderly individuals or those with mobility impairments [4]. Such systems can automatically adjust environmental settings based on users’ gait changes, including modifications to lighting intensity and temperature to enhance comfort and safety.

Foot motion-based biometric technologies have also been widely investigated and applied in areas such as authentication, security certification, and criminal investigations. This is attributed to the unique walking patterns exhibited by different individuals, which are influenced by factors such as weight, height, muscle consumption, tendon and bone conditions, the center of gravity during ambulation, and bone density [4,5,6]. Classifiers in machine learning, including k-nearest neighbors (k-NNs), logistic regression (LR), decision trees (DTs), random forests (RFs), support vector machines (SVMs), and naive Bayes (NB), were demonstrated to possess the capability to recognize gait phases. The training and testing datasets, constructed from the standing and swinging phases of gait, had a significant impact on the classifiers. Additionally, the choice of training methods considerably influenced the performance of the algorithms [7]. In gait feature measurement technologies, visual methods combined with deep learning models facilitate three-dimensional imaging of human locomotion, aiding in generating multiple significant features for fine feature extraction in diverse non-overlapping areas. Vision-based systems utilize Kinect sensors to achieve human gait recognition by integrating skeletal gait energy images (SkeGEI) with relative distance and angle features [8,9]. Siamese Denoising Autoencoder networks (Siamese DAE) consisted of an encoder that compressed the input trajectories and a decoder that reconstructed more accurate skeletal trajectories [10]. The study employed the Faster R-CNN model to extract gait features from samples where no objects were carried, demonstrating excellent recognition accuracy [11]. However, due to the substantial computational resources required for processing sequential image data during analysis, there is a considerable demand for hardware resources in computational units [9,10]. Moreover, its accuracy was significantly affected by variations in clothing, carried items, and walking environments.

In contrast, foot pressure measurement systems restrict data collection to specific areas using pressure sensors for data acquisition [12]. Compared to visual systems, floor-based recognition is relatively less affected by changes in subjects’ clothing; thus it maintains stable accuracy across different scenarios [13]. Analyzing foot pressure data through machine learning and deep learning significantly enhances its recognition analysis and predictive capabilities—for instance, utilizing support vector machines (SVM) [14,15] and random forests for classifying gait patterns; employing convolutional neural networks (CNNs) demonstrates superior performance in extracting large-scale spatial feature data from pressure images [16,17]. Few studies have studied the open set recognition in gait identification. One study proposed a convolution prototype network (CPN) architecture that comprised a CNN feature extractor and prototypes for all known classes to improve the unknown rejection performance [18]. Another study integrated structured network architecture that combines CNNs, recurrent neural networks (RNNs), and self-attention mechanisms for classifying subjects belonging to unknown classes. The proposed architecture utilized wireless smart insoles equipped with eight pressure sensors, a 3D accelerometer, and a 3D gyroscope to collect gait features [19].

In previous studies [20], heterogeneous high-density pressure sensing mats were employed to capture raw foot pressure and trajectory data for preprocessing prior to applying deep learning convolutional neural networks. Building on this foundation, this study proposes a classifier developed through convolutional neural networks combined with autoencoders trained on foot pressure data collected from 60 subjects. Among 60 participants, 48 were classified as authorized individuals and 12 as unauthorized. These data are processed into a 64-dimensional feature vector database while incorporating experimental conditions related to the subjects’ carried items to understand the impact of additional weight on gait recognition and successfully develop a personnel gait recognition system.

## 2. Materials and Methods

### 2.1. Foot Plantar Pressure Measurement System

The pressure sensing mat (Flexible thin pressure sensitive sensor, PI Bioelectronics Co., Ltd。, Taichung, Taiwan) consists of three mat units, each measuring 64 cm × 40 cm, resulting in a total length of 192 cm. Each piezoresistive pressure sensor measures 1 cm × 1 cm, with a total of 7680 sensors embedded within the mat. The pressure sensing range of the piezoresistive sensors is from 5 PSI to 80 PSI. The sampling frequency of the pressure sensing mat is set at 40 Hz, and data transmission between the sensing mat and the computer is facilitated using the UART hardware communication protocol. The data format generated by the pressure sensing mat is a two-dimensional matrix of size 192 × 40, where each element is represented by an 8-bit value, corresponding to a range between 0 and 255.

### 2.2. Gait Measurement

A total of 60 participants were recruited from National Yang Ming Chiao Tung University, comprising 17 females and 43 males. The experimental venue was located in the Engineering Building 5 at the Hsinchu campus of National Yang Ming Chiao Tung University. The Institutional Review Board (IRB) of National Yang Ming Chiao Tung University has approved the data collection from participants in this experiment. Each participant provided informed consent in accordance with the policies of the Yang Ming Chiao Tung University Research Ethics Committee (IRB No. NYCU-REC-111-009WE).

During the experiment, participants were required to wear the provided cloth shoes to eliminate variations in pressure data that could arise from differing sole patterns. Shoe sizes were denoted in US sizing, with the following distribution among participants: 2 individuals wore size 3 shoes, 15 wore size 4, 19 wore size 7, and 24 wore size 10.

**Figure 1:**
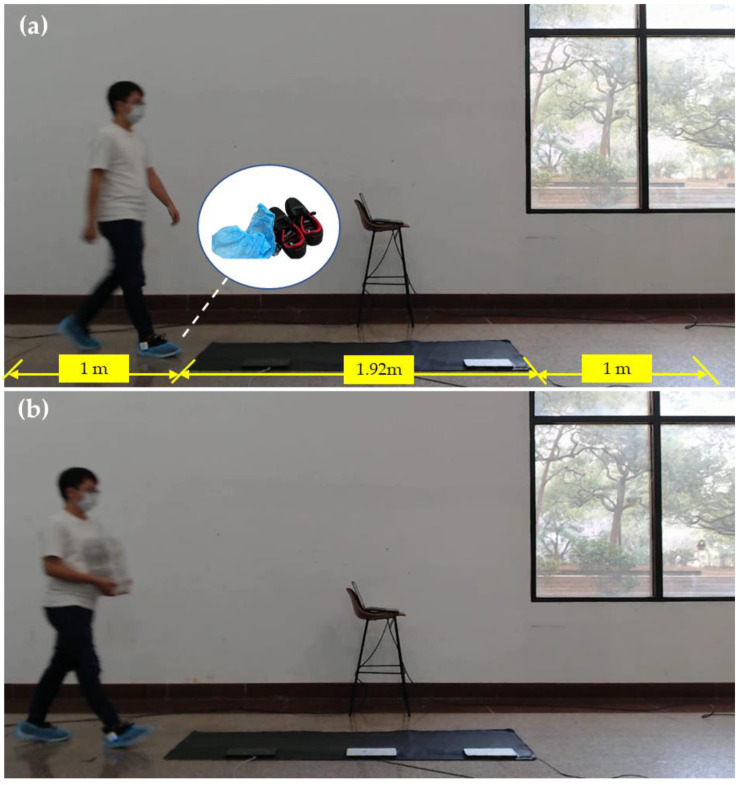
Gait measurement (**a**) Type I trial: walking without carried, (**b**) Type II trial: walking with carried 500 g load.

In the experiment, participants began walking from a point one meter in front of the pressure sensing mat to avoid acceleration effects when starting to walk. The stopping position after passing over the mat was also set one meter from the end of the mat. Throughout the testing process, participants were instructed to walk naturally while maintaining a forward gaze [21]. Each participant was instructed to complete thirty trials of walking at a normal pace on a piezoresistive pressure sensing mat system, where a round trip counted as one trial, as shown in Figure 1a. After completing thirty trials, participants were asked to carry a 500 g object while walking an additional ten trials, as shown in Figure 1b, to observe whether carried additional items would affect recognition rates.

All data will be divided into two types, including Type I trials and Type II trials. The Type I data primarily consists of participants walking on the pressure-sensing mat without any load. The Type II data refers to participants being instructed to walk on the pressure-sensing mat while carrying a 500 g load.

### 2.3. Feature Extraction

**Figure 2:**
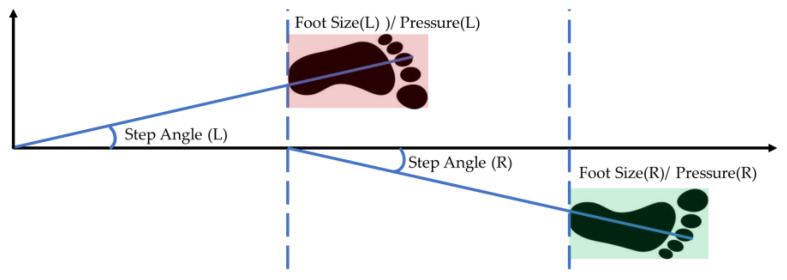
Key features of gait measurement.

The gait measurement parameters in this experiment included three key features: step angle, plantar pressure, and foot size [22], as shown in Figure 2. By utilizing region of interest (ROI) digital image processing techniques [23], the participant’s pressure sensing values recorded during a single traversal of the mat were aggregated.

To reduce the impact of noise on the overall dataset, the gait data were analyzed to detect anomaly points based on their range and distribution. The anomalous points were replaced with the average values of neighboring points. Subsequently, a 32 × 16 mask was employed to segment and outline the pressure distribution areas of the foot to effectively concentrate pressure within the sensing regions, filter out irrelevant data, and facilitate subsequent feature extraction. Then, the features were applied to each point for convolution calculations while simultaneously marking the spatial coordinates along the *x*-axis and *y*-axis to document the footprint area traversed on the mat, which provided valuable spatial information.

Using the *x*-axis and *y*-axis coordinate data from the ROI, continuous observation of this area was maintained, meaning that tracking occurred throughout each gait cycle. When a change in pressure value was detected from zero, the corresponding frame number was marked to indicate the timing of that event. Through this marking, the duration of the gait cycle could be determined, thus yielding temporal information. After combining these two types of information, data filtering was performed to eliminate noise.

Next, the pressure maps for the left foot (32 × 16) and right foot (32 × 16) were merged to create an averaged bilateral feature input image of size 32 × 32. In this study, all input data uniformly utilized the 32 × 32 average pressure map for both feet. This processing approach aided in enhancing data consistency.

To improve the generalization capability of the model, data augmentation techniques were employed to transform the training data, thereby generating additional training samples that contributed to enhancing the model’s generalization ability.

The gait cycle was defined as the duration of movement involving both feet, also referred to as stride length, and encompassed two primary phases: the stance phase and the swing phase. These two phases alternated between each leg. The stance phase constituted approximately 60% of the entire gait cycle, during which the foot remained in contact with the ground, while the swing phase accounted for 40% of the cycle, during which the foot was airborne. Furthermore, each phase consisted of sequences of single support (when only one foot was on the ground) and double support (when both feet were in contact with the ground).

By utilizing the *x*-axis and *y*-axis coordinate data from the region of interest (ROI), continuous observation of the ROI area was maintained, meaning that tracking occurred throughout each gait cycle. When a change in pressure value was detected from zero, the corresponding frame number was marked to indicate the timing of that event. This marking allowed for determining the duration of the gait cycle, thereby providing temporal information.

As previously mentioned, obtaining an average plantar pressure image required both spatial and temporal information [21,24,25]. Initially, participants walked on the sensing mat, followed by summing the pressure values recorded on the mat. Subsequently, spatial information was acquired using ROI techniques. The obtained ROI area was then divided by the frame count based on the gait cycle. By extracting both spatial and temporal information, images sized 32 × 16 pixels were created for each foot. These images were then combined to form a single image of size 32 × 32 pixels, representing information from both feet. This feature image provided insights into pressure distribution, foot angles, and foot sizes.

After calculating the gait cycle for the first step, subsequent steps exhibited varying gait cycles. Therefore, the gait cycle for the second step was calculated, and this process continued until N-1 steps had been analyzed. The number of generated images varied according to each participant’s walking distance; for instance, two average pressure images were produced during three steps, while three average pressure images were generated during four steps. The average footprint images served as input data for the system.

### 2.4. Gait Recognition Model

**Figure 3:**
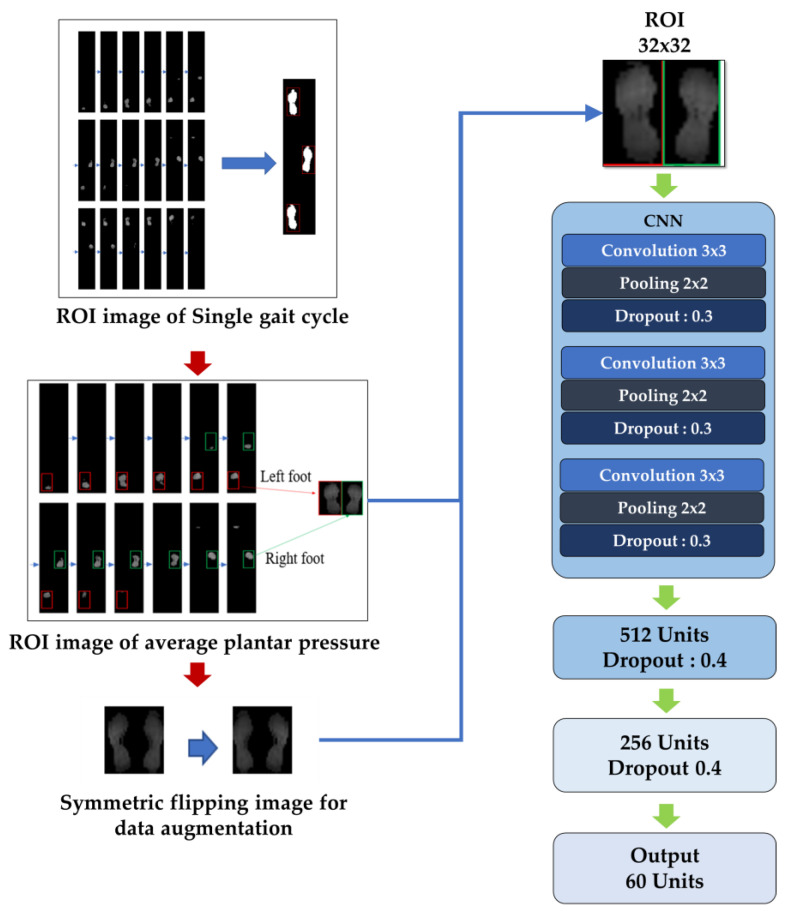
Proposed CNN architecture for gait recognition.

The gait recognition model was divided into two parts. The first part incorporated all 60 participants into the model training to test whether individuals could be identified through average plantar pressure maps. The architecture of the model’s convolutional neural network (CNN) was illustrated in Figure 3, consisting of three convolutional layers and pooling layers, with the output resulting in 60 categories.

The second part involved dividing the 60 participants into 48 known classes and 12 unknown classes. Traditional CNN methods struggled to successfully classify “known and unknown” participants; therefore, thresholds and feature vectors were employed here. If the similarity between the two was below a specified threshold, the data were classified as unknown.

**Figure 4:**
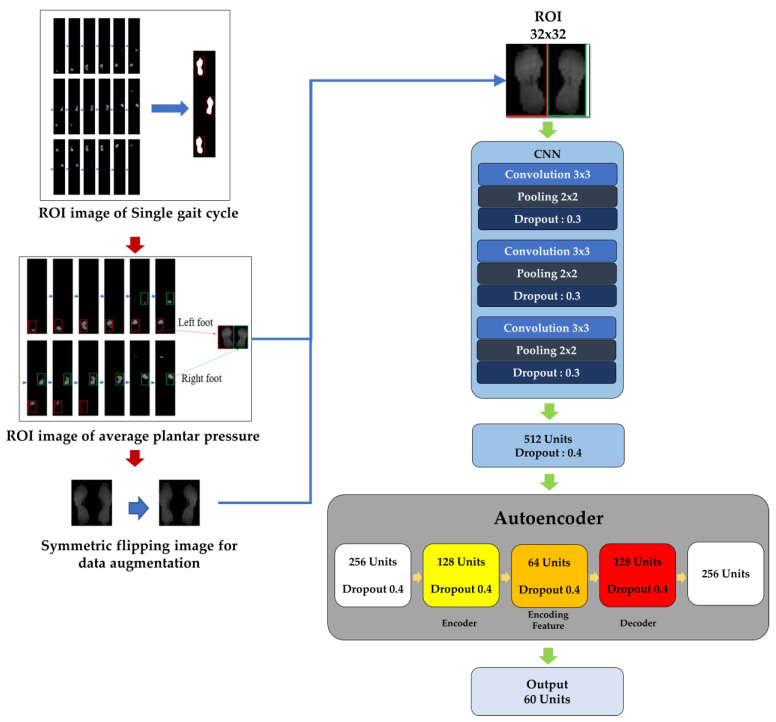
Proposed CNN-AE architecture for gait recognition.

As previously mentioned, the primary advantage of CNN lay in its robust image classification capabilities, while autoencoders (AEs) excelled in feature extraction, dimensionality reduction, and denoising [26]. AE reduced the dimensionality of the data while preserving its fundamental characteristics, enabling a more compact and informative representation of the data [27,28]. Consequently, the model architecture designed in this study integrated the strengths of both CNN and AE to construct an entire deep learning model framework, as depicted in Figure 4.

Initially, input data sized 32 × 32 pixels was fed into the CNN framework. This data underwent feature extraction through three convolutional layers and pooling layers before being flattened and input into a fully connected neural network for training. The output from the previous layer, specifically the hidden layer with 256 neurons, was extracted entirely and subsequently input into the AE framework for unsupervised learning training. Through AE training, feature vectors were effectively reduced to 64 dimensions. Through this deep learning model architecture, CNN performed feature extraction and classification on known data, achieving high classification accuracy. Simultaneously, AE reduced feature vectors to 64 dimensions and stored each individual’s feature vector in a database. The input data, sized 32 × 32 pixels, was entered into the CNN framework; after passing through three convolutional layers and pooling layers for feature extraction, it was then flattened and input into a fully connected neural network for training. The output from the previous layer with 256 neurons was extracted entirely and used for unsupervised learning training within the AE framework [23]. Through AE training, feature vectors were effectively reduced to 64 dimensions.

### 2.5. Dataset

**Figure 5:**
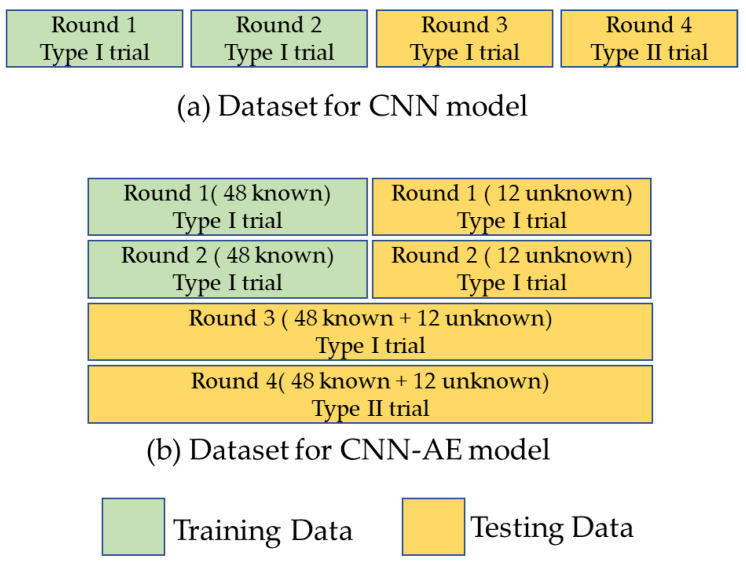
Training Data and Testing Data.

The gait recognition model was divided into two parts as shown in Figure 5. The first part focuses on all 60 categories of participants, utilizing only CNN classification to determine whether average plantar pressure maps could serve as feature maps. The Type I data from the first and second rounds were used for model training data, while the data from the third round of Type I and the fourth round of Type II were designated for testing data.

To expand the application domain of the gait recognition model developed in this study, the 60 participants were divided into 48 known subjects and 12 unknown subjects to evaluate the system’s performance in distinguishing between known and unknown individuals. The training process began with classification using a convolutional neural network (CNN), followed by dimensionality reduction in the feature vectors through an autoencoder (AE). Ultimately, all feature vectors were stored in a database. The third-round data from the Type I trial of known subjects and data from the Type II trial were also used as additional testing data.

In each trial, subjects produced 40 unit steps. The convolutional neural network (CNN) model included data from 60 subjects, with both the training and testing datasets comprising 4800 unit steps. In the CNN-autoencoder (CNN-AE) model, the training dataset consisted of 3840 unit steps generated by 48 known category subjects, while the remaining data constituted the testing dataset, totaling 5760 unit steps.

### 2.6. Database

Through the aforementioned deep learning model architecture, the convolutional neural network (CNN) can perform feature extraction and classification on known data, thereby achieving high classification accuracy. Simultaneously, the autoencoder (AE) is capable of reducing feature vectors to 64 dimensions, with each individual’s feature vector stored in a database. Once each data entry was saved to the database, the 64-dimensional feature vectors underwent standardization. The formula used for this process is given in the equation:

(1) $${{\overset{⃑}{x}}^{\prime }}_{i}=\frac{{\overset{⃑}{x}}_{i}-\mu_{i}}{\sigma_{i}}$$

where, ${\overset{⃑}{x}}_{i}$ represents the original 64-dimensional vector for a specific data entry, which is adjusted by subtracting the mean $\mu_{i}$ and dividing by the standard deviation $\sigma_{i}$. This process enhances the conformity of the feature vectors to a normal distribution and reduces the influence of outliers. Standardization is beneficial in eliminating scale effects within the data, allowing for comparability among variables with different scales or units. Additionally, it aids in improving the efficiency of data processing and analysis.

Subsequently, using the newly standardized feature vector ${{\overset{⃑}{x}}^{\prime }}_{i}$, cosine similarity is computed between the known feature vectors and those of interest. This calculation measures the similarity between two feature vectors, yielding a value within the range of −1 to 1, as described in Equation (2):

(2) $$Similarity=\cos\left(\theta \right)=\frac{A\cdot B}{\left\|A\right\|\left\|B\right\|}$$

In this context, *A* and *B* are two vectors, where “·” denotes the dot product of the vectors and “‖‖” signifies their Euclidean lengths. Mathematically, cosine similarity quantifies how similar two vectors are based on the cosine of the angle between them. It is widely used in various fields such as vector space models, text classification, image processing, and machine learning.

### 2.7. Evaluation Metrics

(1) False Rejected Rate (FRR)

A false rejection occurs when a biometric system incorrectly classifies an authorized individual as unauthorized [29]. This type of error is commonly referred to as a Type I error. False rejections may lead to user frustration, economic losses, and expenditure to re-authenticate authorized users.

(3) $$FRR=\frac{FN}{FN+TP}$$

where FN (false negative) is the number of events when the system incorrectly rejects the data. TP (true positive) is the number of events that the system correctly identifies an authorized individual as legitimate. FN + TP indicates the total number of events.

(2) False Acceptance Rate (FAR)

A false acceptance occurs when an unauthorized individual is incorrectly recognized as valid by a biometric system [23]. This type of error is commonly referred to as a Type II error. When an organization experiences a high rate of false rejections, it may be compelled to lower the system’s accuracy by reducing the amount of data collected during the authentication process. Such a reduction in data points increases the risk of elevating the false acceptance rate, thereby exposing the organization to the potential threat of unauthorized users gaining access to secure areas or information.

(4) $$FAR=\frac{FP}{FP+TN}$$

where FP (false positive) is the number of events when the system incorrectly rejects the data. FP + TN indicates the total number of unauthorized events.

(3) Equal Error Rate (EER)

The equal error rate (EER) serves as a pivotal performance metric within biometric systems, defined as the point at which the false acceptance rate (FAR) is equal to the false rejection rate (FRR) [23]. This intersection signifies the thresholds at which the probability of incorrectly accepting an unauthorized individual as legitimate corresponds with the probability of erroneously rejecting an authorized user. A lower EER indicates a more precise biometric system, reflecting an optimal balance between security and usability.

(4) Receiver Operating Characteristic (ROC) curve

The ROC curve [30] is a graphical representation utilized to assess the performance of a binary classifier system as its discrimination threshold varies. The ROC curve serves as a tool for evaluating the trade-offs between sensitivity and specificity, thereby providing insights into the diagnostic capabilities of the classifier. A salient feature of the ROC curve is its ability to illustrate how alterations in the threshold influence the rates of true positives and false positives, facilitating a comprehensive evaluation of model performance across diverse operating conditions. The area under the ROC curve (AUC) is frequently employed as a singular metric to summarize the overall performance of the classifier, with higher values indicative of superior discriminative ability. The ROC curve and AUC are extensively utilized across various disciplines, including medicine, machine learning [31], and signal detection theory, to effectively compare different diagnostic tests or models. This analytical approach allows researchers and practitioners to optimize classification thresholds, ultimately enhancing decision-making processes in clinical and technical applications.

(5) Accuracy

Accuracy is a straightforward metric that reflects the overall predictive performance of a model across all samples. However, in cases of class imbalance, accuracy may provide misleading results [23]. Accuracy is the proportion of correctly predicted samples out of the total number of samples. Its mathematical formula is:

(5) $$Accuracy=\frac{TP+TN}{TP+FP+FN+TN}$$

(6) Precision

Precision measures how many of the predicted positive results are truly positive. The metric is particularly useful in applications where the cost of false positives is high, such as spam email classification [23]. Precision refers to the proportion of actual positive samples among all samples predicted as positive by the model. Its mathematical formula is:

(6) $$Precision=\frac{TP}{TP+FP}$$

(7) Recall

Recall measures how well a model can detect actual positive samples and is especially important in applications that require a high detection rate, such as disease screening [32]. Recall, also known as true positive rate (TPR), is the proportion of actual positive samples that are correctly classified as positive. Its mathematical formula is:

(7) $$Recall=\frac{TP}{TP+FN}$$

(8) F1-Score

The F1 score [33] is particularly useful when dealing with imbalanced datasets because it considers both precision and recall, providing a comprehensive evaluation of model performance. The F1 score is the harmonic mean of precision and recall, aiming to balance these two metrics. Its mathematical formula is:

(8) $$F1=\frac{2\times Recall\times Precision}{Recall+Precision}$$

## 3. Results

**Figure 6:**
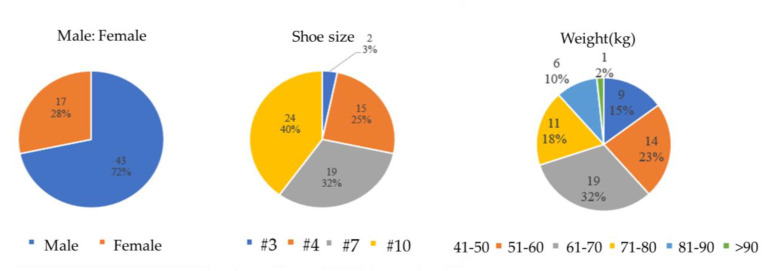
The feature distribution of participating subjects.

This experiment involved a total of 60 participating subjects, comprising 17 females and 43 males. For this study, fabric shoes were provided to the participants to control for variables, as they might otherwise wear shoes with different sole patterns. Shoe sizes were indicated using US sizing. The distribution of participating subjects according to their shoe sizes is as follows: 2 participants wore size 3, 15 participants wore size 4, 19 participants wore size 7, and 24 participants wore size 10. The gender ratio of the participating subjects, along with the distribution of shoe sizes and body weights, was illustrated in Figure 6.

### 3.1. Known Classes Testing

**Figure 7:**
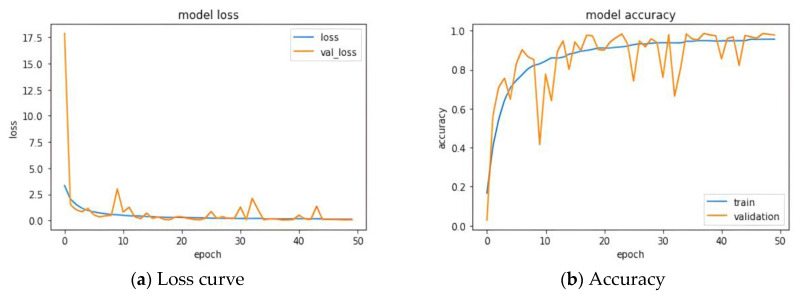
CNN model performance: (**a**) Loss curve and (**b**) Accuracy.

The data from all 60 participating subjects were classified using a CNN for accuracy testing to evaluate the effectiveness of utilizing average plantar pressure maps as feature maps. During model training, the gait data from the first and second rounds of the Type I trial were used as training data, while the testing data consisted of the gait data from the third round of the Type I trial and the data from Type II. The model parameters were set with a learning rate of 0.001, an epoch count of 50, a batch size of 16, and a validation split of 0.1. Figure 7 illustrates the loss and accuracy during the training of the model. As shown in the figures, the training of the entire model tends to converge around epoch 20 [28].

**Figure 8:**
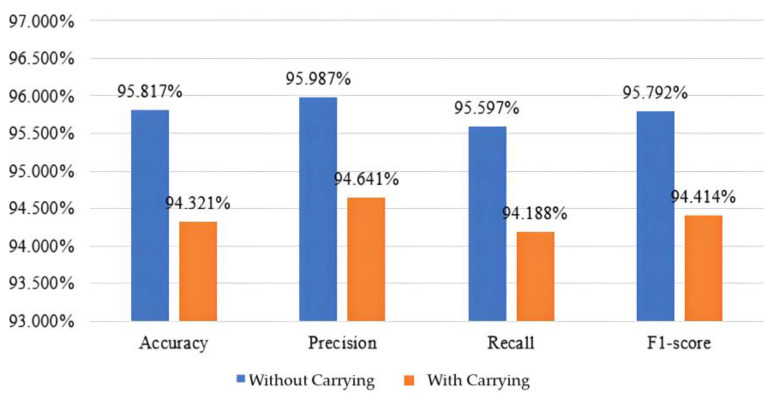
The performance of the CNN model.

Based on the aforementioned metrics, the performance of the model was calculated. As shown in Figure 8, under the condition of no load, the accuracy reached 95.817%, with a precision of 95.987%, a recall of 95.597%, and an F1 score of 95.792%. Conversely, under the condition of carrying a load, the accuracy was 94.321%, with a precision of 94.641%, a recall of 94.188%, and an F1 score of 94.414%. These metrics indicate that the model successfully classified the data using average plantar pressure maps as input. Notably, even without training the model under load conditions, satisfactory performance was achieved during testing, demonstrating the robustness of the system across varying conditions.

### 3.2. Unknown Classes Testing

The 60 participants were divided into 48 known subjects and 12 unknown subjects to evaluate the system’s performance in distinguishing between known and unknown individuals. The randomly selected participant numbers for the unknown subjects were: 2, 4, 10, 11, 20, 24, 46, 50, 51, 55, 56, and 57. Participants with these numbers were designated as unknown subjects, while the remaining participants were considered known subjects and were used for model training with data from the first and the second rounds of the Type I trial. The training process began with classification using a convolutional neural network (CNN), followed by dimensionality reduction in the feature vectors through an autoencoder (AE). Ultimately, all feature vectors were stored in a database. The third round of data from the Type I trial of the known subjects was utilized as testing data for validation. Finally, data from the Type II trial were also employed as additional testing data. This experimental design allows for an effective assessment of the system’s performance in distinguishing between known and unknown subjects, further investigating the feasibility and benefits of the system in practical applications.

**Figure 9:**
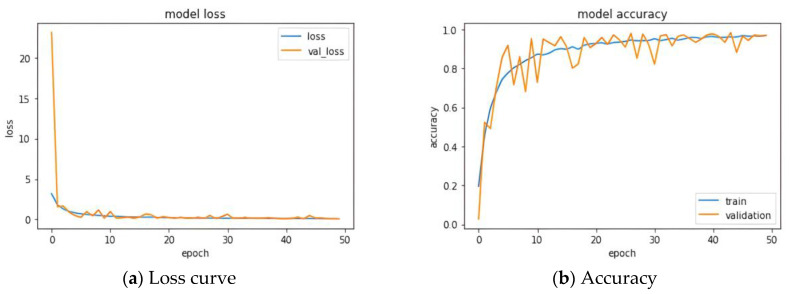
CNN-AE model: (**a**) Loss curve and (**b**) Accuracy.

The model parameters were configured with a learning rate of 0.001, an epoch count of 50, a batch size of 16, and a validation split of 0.1. Figure 9 presents the loss and accuracy during the training of the model. As indicated in these figures, the training of the entire model converges around epoch 20 [34].

**Figure 10:**
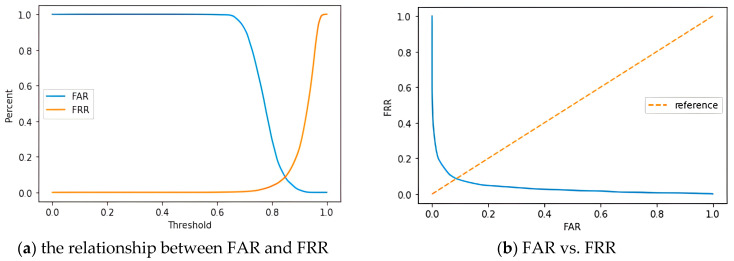
CNN-AE model: (**a**) the relationship between FAR and FRR, and (**b**) FAR vs. FRR.

In the Type I trial, an evaluation was conducted on both known and unknown subjects. As illustrated in Figure 10a, the false acceptance rate (FAR) and false rejection rate (FRR) for both groups can be observed. The intersection of these two rates represents the Equal Error Rate (EER). By setting the threshold value at 0.85, the balance point between FAR and FRR is achieved at an EER of 8.55%. This configuration allows the system to attain a lower balance between FAR and FRR, thereby enhancing the reliability and performance of the system. In Figure 10b, the false acceptance rate (FAR) was plotted on the *x*-axis, while the false rejection rate (FRR) was plotted on the *y*-axis, with adjustments made according to different threshold values. This figure illustrated the system’s performance under varying thresholds, with the diagonal line serving as a reference line. The intersection of this reference line with the plotted lines indicated the Equal Error Rate (EER). This graphical representation allowed for a clearer observation of the system’s balance at different thresholds, further enabling the assessment of model performance. Through fine-tuning of the thresholds, an appropriate balance between FAR and FRR was achieved, thereby enhancing the system’s reliability and practicality.

**Figure 11:**
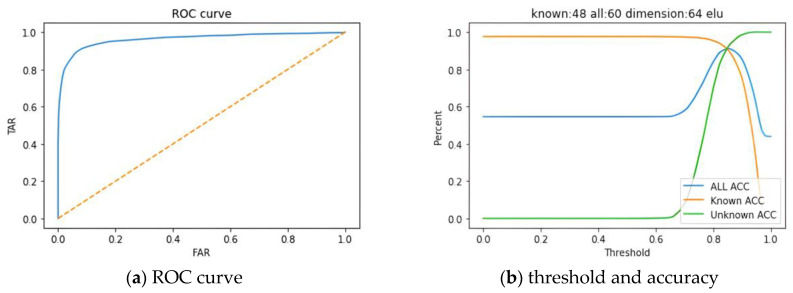
CNN-AE model (**a**) ROC curve and (**b**) threshold and accuracy.

Figure 11a illustrated the ROC curve with the false acceptance rate (FAR) plotted on the *x*-axis and the true acceptance rate (TAR) on the *y*-axis. The area under the curve (AUC) was calculated to be 0.9629, indicating that the system exhibited strong classification capabilities. In Figure 11b, it was shown that as the threshold gradually increased, the accuracy of unknown subjects also rose. However, it was necessary to identify an appropriate threshold to balance the accuracy between known and unknown subjects. In this experiment, the threshold was adjusted to 0.85, as previously mentioned, which corresponded to the location of the EER. This adjustment allowed for a relatively balanced state between the accuracies of known and unknown subjects. Furthermore, when the threshold was set at 0.85, the accuracy for known subjects reached 90.83%, while the accuracy for unknown subjects was recorded at 91.71%. The overall accuracy (for both known and unknown subjects) was determined to be 91.22%. This balanced setting enhanced the system’s precision in distinguishing between known and unknown individuals.

**Figure 12:**
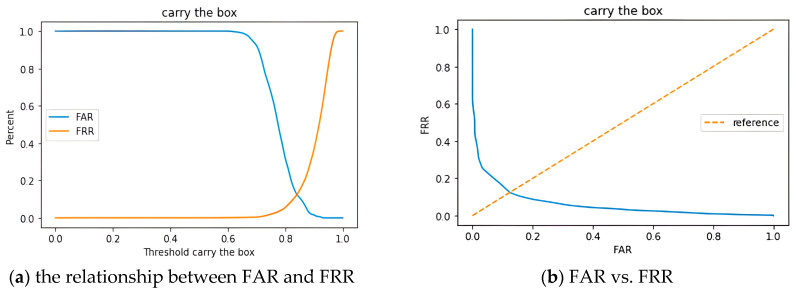
CNN-AE model: (**a**) the relationship between FAR and FRR and (**b**) FAR vs. FRR.

In the Type II evaluation, both known and unknown subjects were assessed. As shown in Figure 12a, the false acceptance rate (FAR) and false rejection rate (FRR) for authorized and unauthorized individuals were observed. The intersection of these two rates represented the equal error rate (EER). The threshold value was set at 0.84, at which point the balance between FAR and FRR was achieved, resulting in an EER of 12.37%.

**Figure 13:**
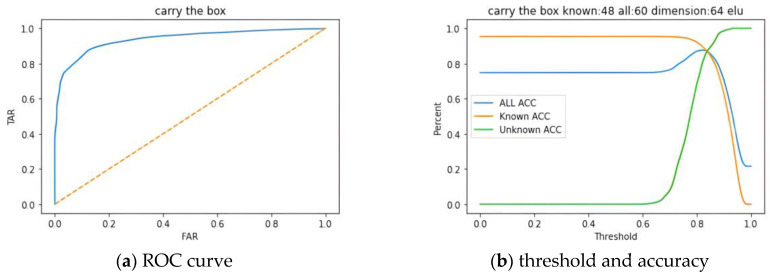
CNN-AE model of type II trial. (**a**) ROC curve, (**b**) threshold and accuracy.

Figure 12b displayed a graph with FAR on the *x*-axis and FRR on the *y*-axis, adjusted according to various threshold values. This graph illustrated the system’s performance under different thresholds, with the diagonal line serving as a reference line. The intersection of this reference line with the plotted lines indicated the EER. This graphical representation allowed for a clearer observation of the system’s balance at different thresholds, further facilitating the assessment of model performance. Through fine-tuning of the thresholds, an appropriate balance between FAR and FRR was achieved, thereby enhancing the system’s reliability and practicality. Figure 13a depicted the ROC curve with the FAR plotted on the *x*-axis and the true acceptance rate (TAR) on the *y*-axis. The area under the curve (AUC) was calculated to be 0.9375, indicating that the system exhibited good classification capabilities. In Figure 13b, it was shown that as the threshold gradually increased, the accuracy of unauthorized personnel also rose. However, it was necessary to identify an appropriate threshold to balance the accuracy between authorized and unauthorized personnel. In this experiment, the threshold was adjusted to 0.84, corresponding to the location of the EER. This adjustment resulted in a relatively balanced state between the accuracies of authorized and unauthorized personnel [35].

Furthermore, when the threshold was set at 0.84, the accuracy for authorized personnel reached 86.71%, while the accuracy for unauthorized personnel was recorded at 87.55%. The overall accuracy (for both authorized and unauthorized personnel) was determined to be 86.89%. This balanced setting enhanced the system’s precision in distinguishing between authorized and unauthorized individuals.

**Figure 14:**
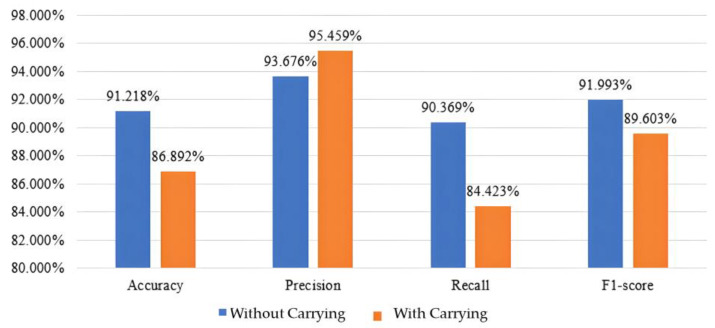
Evaluation metrics results of CNN model.

As shown in Figure 14, when no load was held, the accuracy reached 91.218%, with a precision of 93.676%, a recall of 90.369%, and an F1 score of 91.993%. In contrast, when carrying a load, the accuracy was recorded at 86.892%, with a precision of 95.459%, a recall of 84.423%, and an F1 score of 89.603%. The higher precision indicated that the model was more cautious and accurate when determining samples as positive, suggesting that it only provided positive judgments under highly certain conditions, thus avoiding excessive false positives.

On the other hand, the lower recall suggested that authorized individuals were more likely to be misclassified as unauthorized due to training data being limited to scenarios without a load; thus, judgments involving carrying a load were affected. In specific contexts where false positives (misclassifying unauthorized individuals as authorized) carried high costs or required high certainty for positive determinations, this trade-off is critical.

However, it is important to note that high precision may lead to a reduced recall since a more cautious model may miss some true positives. In certain application scenarios, this trade-off may be acceptable.

## 4. Discussion

Table 1 listed all cases of misclassification in the CNN model of subjects who were not carrying the load. These misclassifications were related to identical shoe sizes, with only one group of subjects having shared the same weight. Therefore, weight appeared to be one factor that contributed to the misclassifications. Moreover, variations in foot pressure distribution during walking or differences in foot angles might have also played a significant role in causing these misclassifications.

Table 2 presented cases of misclassification in the CNN model for subjects carrying a load. The number of misclassifications was greater than that observed when subjects were not carrying a load. This increase was attributed to the consideration that, in practical applications, the weight of the load held by individuals could vary. Consequently, the CNN training dataset utilized only data from subjects who were not carried a load, and the conditions involving load-carried were not incorporated into the model training. Therefore, the data from subjects carrying a load was considered relatively new for the CNN model. An examination of Table 2 revealed that all misclassifications were associated with identical shoe sizes. However, unlike the previous situation, three groups of subjects had similar weights in this instance. This could have resulted from changes in the center of gravity while walking with the load, leading to variations in foot pressure distribution or alterations in foot angles, which caused misclassifications among subjects with similar weights. Notably, the three subjects with the highest number of misclassifications did not have similar weights. This indicated that weight was merely one factor contributing to the misclassifications and that errors were also related to variations in foot size, angle, or pressure distribution.

Standardization played a crucial role in the preprocessing of the CNN-AE model. The recognition results showed that after all feature vectors were standardized, the equal error rate (EER) value was further reduced, decreasing from an initial EER of 9.84% to 8.55%.

This study employs several preprocessing techniques, including region of interest (ROI) selection and feature image extraction, along with methods such as horizontal flipping and standardization to address the issue of overfitting. Additionally, it considers the effects of lightweight objects carried by subjects. A summary of the performance comparison is listed in Table 3. Although 3D-CNN models demonstrate superior recognition rates, their substantial computational requirements significantly hinder real-time recognition and computational efficiency. This study indicates that for subjects carrying objects, increased load results in uneven pressure distribution on the foot, thereby diminishing recognition accuracy. Consequently, gait features encounter stability challenges under varying load conditions, which also presents a critical avenue for model improvement.

## 5. Conclusions and Future Work

In this paper, the application of an autoencoder to further enhance the features extracted by the CNN has been demonstrated in this work. This approach offered several advantages: (1) to provide a more representative and condensed feature space; (2) to eliminate noise and redundant information, thereby improving model stability; and (3) to complement the shortcomings of CNN at the feature hierarchy level, achieving the integration of local and global features.

We collected data from a total of 60 subjects and divided it into two parts: the first part involved classifying all 60 categories, while the second part classified 48 subjects as known subjects and the remaining 12 as unknown subjects. In the first part, classification was performed solely using a CNN deep learning approach. When subjects were not carrying the load, the model achieved an accuracy of 95.817%, precision of 95.987%, recall of 95.597%, and an F1-score of 95.792%. In contrast, when subjects were carrying the load, the accuracy decreased to 94.321%, with precision at 94.641%, recall at 94.188%, and an F1-score of 94.414%. This confirms the effectiveness of using average foot pressure maps as input images. In the second part, both CNN-AE architectures were utilized to convert the average foot pressure maps into 64-dimensional feature vectors, followed by threshold-based classification to determine whether subjects were authorized or unauthorized personnel. In scenarios where subjects were not carrying the load, the EER was recorded at 8.55%, with an accuracy of 91.218%, precision of 93.676%, recall of 90.369%, and an F1-score of 91.993%. This indicates that the model successfully identified a majority of actual positives. However, when subjects were carrying the load, there was a decline in model performance due to the training data comprising only instances without the load. Nevertheless, the system demonstrated robustness, with an EER of 12.37%, accuracy reaching 85.648%, precision at 94.459%, recall at 84.423%, and an F1-score of 89.603%.

The experimental results indicate that varying the weight of the handheld load significantly affects gait recognition outcomes. The proposed system utilizing a pressure sensing mat with a CNN-AE architecture demonstrates the potential to recognize load effects, providing an alternative to personal wearable pressure insoles or inertial measurement units (IMUs) and serving as a viable option for public spaces or medical equipment. In future research, incorporating additional feature parameters for the dataset, such as different load weights, body mass index (BMI), and clothing weight, will enhance the recognition model regarding load effects, thereby improving the applicability of the model.

## Figures and Tables

**Table 1:** Misclassification in the CNN model for subjects was not carried the load.

Misclassification Count	Actual Number	Predicted Number	Actual Weight	Predicted Weight	Actual Shoe Size	Predicted Shoe Size	Common Points
**5**	55	51	75	75	7	7	Shoe Size, Weight
**3**	29	20	60	73	10	10	Shoe Size
**3**	46	39	75	93	10	10	Shoe Size
**3**	55	54	75	57	7	7	Shoe Size

**Table 2:** Misclassification in the CNN model for subjects carried the load.

Misclassification Count	Actual Number	Predicted Number	Actual Weight	Predicted Weight	Actual Shoe Size	Predicted Shoe Size	Common Points
**11**	46	39	75	93	10	10	Shoe Size
**9**	1	12	85	68	10	10	Shoe Size
**8**	5	31	47	62	4	4	Shoe Size
**7**	55	51	75	75	7	7	Shoe Size, Weight
**4**	16	43	63	66	10	10	Shoe Size, Weight

**Table 3:** Performance comparison of gait recognition methods.

Framework	Author	Sensor System	Model	Subjects	Result
**Closed set**	Costilla-Reyes et al. [34]	Plastic optical fiber	3D-CNN	13	Without carried object: 97.88%
	Chiou et al. [20]	Piezoresistive sensors	CNN	30	Without carried object: 92.08%
	This work	Piezoresistive sensors	CNN	60	Without carried object: 95.8% With carried object: 94.23%
**Open set**	Moon et al. [18]	Smart insole	CNNs, RNNs and self-attention	40	Without carried object: 95.3%
	This work	Piezoresistive sensors	CNN-AE	48 known and 12 unkown	Without carried object: 91.218% With carried object: 85.648%

## Data Availability

The data presented in this study are available upon request from the corresponding author.
